# Life Profile of Vaidyan Puthiyedathu Raman Menon

**DOI:** 10.1016/j.jaim.2023.100861

**Published:** 2024-01-21

**Authors:** V.J. Rahul, P.V. Ramadas, P. Nair Leena, C. Ushakumari

**Affiliations:** Department of Maulika Siddhanta (Basic Principles of Ayurveda), Amrita School of Ayurveda, Amrita Vishwa Vidyapeetham, Amritapuri, India

## 1 Introduction

Vaidyan Puthiyedathu Raman Menon (1877–1965) was an Ayurveda practitioner and scholar from Kerala, who is known as a critical commentator of Ashtanga Hridayam and an author. He is popularly known for his two literary works, Sararthabodhini Commentary on Ashtanga Hridayam and Śirassekādi Vidhi. His contributions are considered unique as one of them is a critical commentary to a classical text based on personal clinical experiences and the other is intended to provide standardized methods for therapeutic procedures of Ayurveda.

In major Ayurveda textbooks, certain aspects are found to have only minimal explanations. For example, *abhyanga* (oil massage) is mentioned in texts but there are no detailed explanations of positions, direction of application, duration etc. With clinical experience and evidence-based practice, Vaidyan Raman Menon could establish clear, scientifically sound explanations of *snehana* (oil application) and *svedana* (sudation) procedures, along with the right positions and methods in which these were to be done which are altogether popularly branded as *‘Keraliya Panchakarma/Chikitsa vidhi*). Popularizing such contributions and the contributors of such works will be a great honour to such eminent personalities of the science.

Among the classical texts of Ayurveda, Ashtanga Hridayam always held an important status in Kerala, both in the clinical and academic sectors. Literary works based on Ashtanga Hridayam have been written by many authors from Kerala, some of which are commentaries like Sasilekha by Indu, Kairali by Pulamanthol Mooss, *Vakyapradipika* by Alattiyur Parameswaran Nambudiri, Saratha Darpanam and Bhavaprakasam by Kaikulangara Rama Variar, Bhaskaram by Uppottu Kannan, Arunodayaṃ by Kayikkara Govindan Vaidyan, Vasudeviyamṃ by Vasudeva Sharma, Hridayabodhika by Sridasa Pandita, original works based on subject matter of Ashtanga Hridayam like Hridayapriya and Sukhasadhakam by Vaikath Pachu Moothathu. [1]. These works are proof for the contributions from the scholars of Kerala in the detailed study and updating of Ashtanga Hridayam.

## 2 Personal profile

Vaidyan Puthiyedathu Raman Menon was born in 1877 to Meykkattumanaykkal Narayanan Nambutiri and Puthiyedathu Paruvamma in Poyya village of Thrissur district in Kerala. After primary education under his father, he went to the famous ‘Kodungalloor *Pathshala* (also known as the Kodungalloor Gurukulam or Kovilakam) for higher education under Kunjan Thamburan. At the age of 20 years, he returned home without completing his education, since, according to his horoscope, his lifespan was predicted to be 20 years. After one year of prayers and rituals, he resumed his medical education under ‘Kavisarvabhauman’ Kochunni Thamburan (the younger brother of Kunjan Thamburan). [2]

His Ayurveda education was primarily based on Ashtanga Hridayam Sarvanga Sundarī commentary, along with Ashtanga Sutrasthanam, Charaka Samhita, Sushruta Samhita, Sharangadhara Samhita, Madhava Nidanam and Rasaratna Samucchayam. He learned Ayurveda initially from his father Meykkattumanaykkal Narayanan Nambutiri. He also gained practical knowledge on treatment procedures and medicine preparation under the royal physician, Eechara Varier. After 12 years of education, he returned home and started his medical practice. [2]

**Fig. 1:**
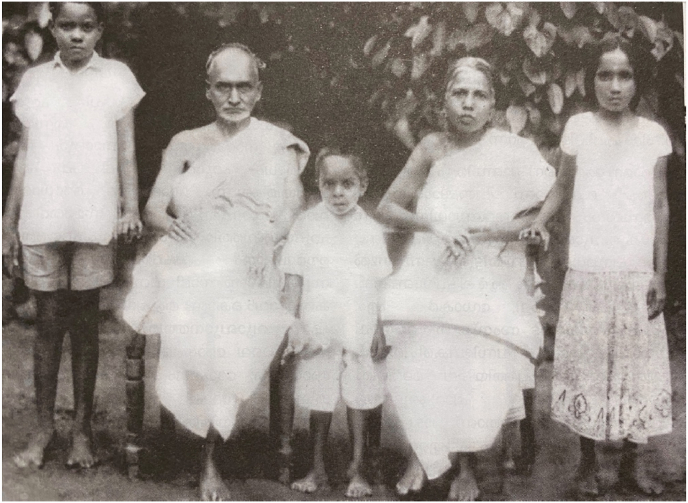
Vaidyan Raman Menon with wife and grandchildren.

He came to be known as an eminent practitioner, especially in ‘Jvaracikitsa’, who also questioned the methods of several widely-followed therapeutic procedures that were practiced at the time, but which did not have scientific standardizations. His clinical practice was primarily in Pooppathy, Thrissur district, Kerala, although he was also known to have been invited for treatment to different parts of Kerala. He was also keen in scientific writing, and several of his articles were said to be published from a monthly publication from Bombay (present-day Mumbai), the details of which are not known. There are records of unpublished literary works attributed to the author preserved as manuscripts at his ancestral home (Fig. 1). [3]

His wife was Thaazhathuveettil Kunjikuttiyamma. He had 4 children, two daughters and two sons. Although none of them were known to have practiced Ayurveda, the oldest among them, Smt. Gauriyamma, who was also a Sanskrit scholar, was believed to have learnt *balachikitsa* from Vaidyan Raman Menon. The youngest among them, Smt. Parukkuttiyamma was the person who preserved the original manuscripts of his works.[2] Vaidyan Puthiyedathu Raman Menon passed away at the age of 88 in the year 1965.

## 3 Author's writing style

Generally, most commentaries to classical Ayurveda texts provide explanations to selected terms of the original text, explain complex concepts, and provide justifications to the matter in the text, without critically evaluating them. But rarely in some commentaries, the authors also try to critically evaluate the classical texts, and provide their explanations and interpretations which might be different to the generally accepted ones. Sararthabodhini by Vaidyan Puthiyedathu Raman Menon is one such critical commentary to Ashtanga Hridayam Sutrasthanam.

In the preface of the book Śirassekādi Vidhi, in the author himself mentions the reasons for writing the book. According to him, practitioners of Kerala were performing procedures like *dhara* (pouring fluids over the whole body or parts of the body as a continuous stream), *śirasseka* (pouring fluids on the head) etc. without following standardized methods since the Samhitas did not explain these improvised therapies in detail. He adds that books like ‘Dhārākalpaṃ’, that explained some therapeutic procedures, were not completely in agreement with the fundamental principles of Ayurveda. So, after consulting with his well-experienced teachers, the author prepared this book detailing scientifically-supported procedures and their logics based on fundamental Ayurveda principles, focusing on the system followed by the ‘Ashtavaidyas’ of Kerala. [2]

A preface was written to the first edition of Śirassekādi Vidhi by ‘*Kottaram Vaidyan*’ K. V. Krishna Varier, in which he says that at the time of writing the book, such an effort to present standardized methods for Ayurveda therapies was commendable. He opines that having standard methods increases safety of the procedures of Ayurveda since different *vaidyas* of the time performed these therapies in different ways, which was not ideal. He also mentions the superiority of Śirassekādi Vidhi over the book ‘Dhārākalpaṃ’ which seemed to contradict with some principles of classical Ayurveda. [2]

## 4 Literary contributions

### 4.1 Sārarthabodhini Commentary on Ashtanga Hridayam (Fig. 2)

The Sārārthabodhini Commentary was originally written in Sanskrit, using Malayalam script. Additions, deletions and substitutions to shlokas can be found in the text, while certain explanations given in the commentary follow an approach different from most other commentators.

**Fig. 2:**
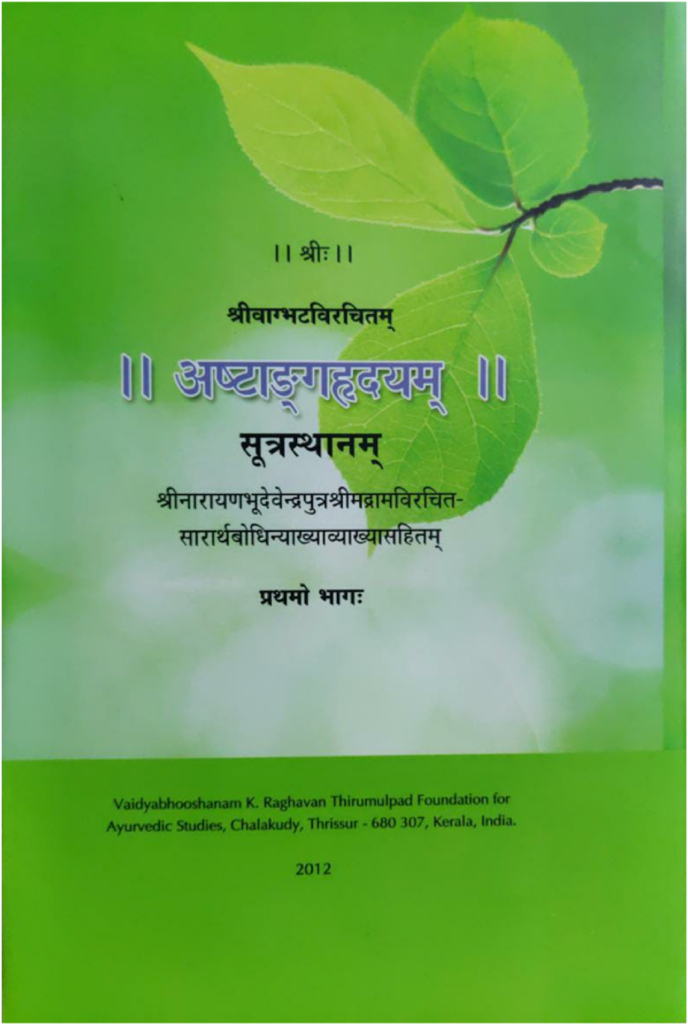
Cover page of Sararthabodhini Commentary on Ashtanga Hridayam In this version of Ashtanga Hridayam Sutrasthanam, the author - Vaidyan Raman Menon - edits some of the original shlokas, and interprets certain portions different to other popular commentaries, which are not common practices in Ayurveda.

The original paper manuscript was preserved by the family of the author. Nearly 50 years after the death of the author, the work was rewritten in Devanagari script, and the first 15 chapters were published by Vaidyabhooshanam K. Raghavan Thirumulpad Foundation (VKRTF) in the year 2012, edited by Dr. M. Prasad. There is a mention of the commentary in a book called ‘Hereditary Physicians of Kerala - Traditional Medicine and Ayurveda in Modern India’, authored by Indudharan Menon, by Routledge Publication, New York. According to this reference, this commentary is believed to be the last known commentary to Ashtanga Hridayam written in Sanskrit.[4]

In Sārārthabodhini, the author also presents his logic for some of the changes he has made to the text. Changes were made to some parts based on his own personal clinical experiences, while he has also used references from other Samhitas to make some other changes. Sanskrit grammar rules are also mentioned to suggest grammatical changes. For example, in the context where the original treatise says that every *dravya* (object) in the world can be considered as an *ashadha* (medicine), the author here directly refutes the statement and opines that there may be objects that are not useful to a *vaidya* as a medicine, and uses the examples of mushroom or prawns, which may not be very useful to *vaidyas* in common clinical practice. While medicinal value of every *dravya* may be important in a theoretical or philosophical point of view, here the author prefers to keep the textbook as a practical version for practicing *vaidyas*, for whom every *dravya* may not be equally useful while dealing with patients.

### 4.2 Śirassekādi Vidhi (Fig. 3)

(‘A Handbook on the Principles and Practices of Keraliya Panchakarma’)

**Fig. 3:**
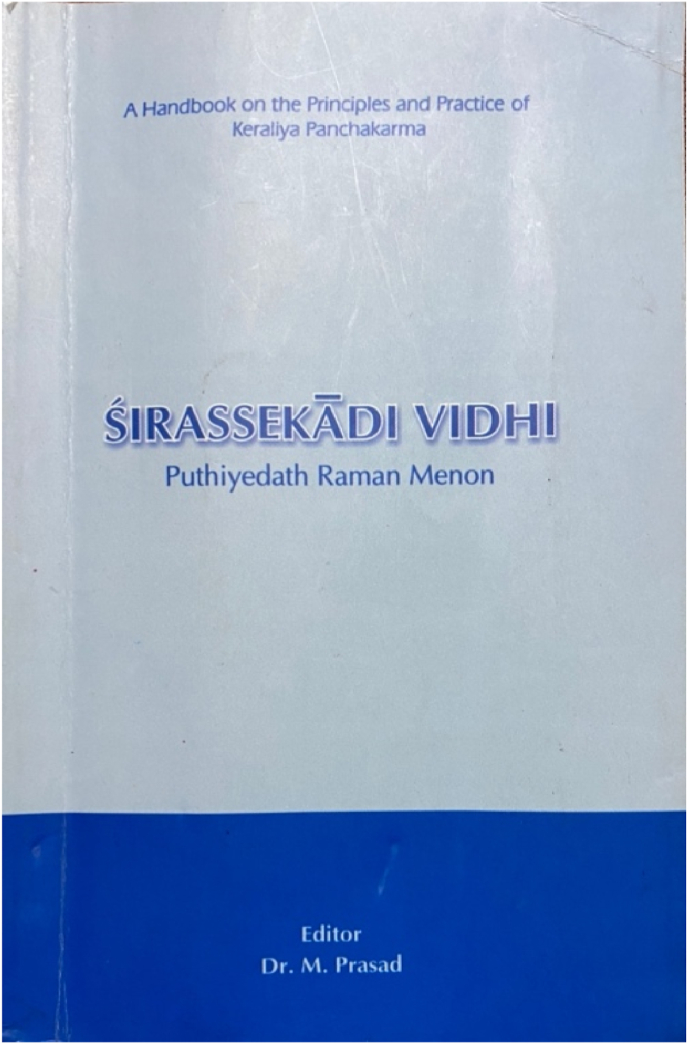
Cover page of Śirassekādi Vidhi.

Śirassekādi Vidhi is, in fact, the first published literary work of Vaidyan Puthiyedathu Raman Menon. This book is an effort to authentically document some of the Ayurveda treatment techniques, which were not detailed in classical Ayurveda texts, but were widely practiced in Kerala.

In the book written in Sanskrit, the author also gives *anvaya* (a logical connection/sequence of words which is different from the poetic usage) to *shlokas* (verses) and explains the *ślokas* using his Malayalam commentary named Bhāvaprabodhini in a simpler and more detailed manner.

The subject in Śirassekādi Vidhi is presented in seven chapters, five chapters explaining one therapeutic procedure each and one chapter each on the suitable time of year for procedures and the *snehapana* (consumption of sneha dravya - fats, oils etc.) to be done before the procedure. Respective chapters detail the method of preparation of medicines, the right quantities of medicines to be used etc. relevant to the procedure explained.

The therapeutic procedures such as *takradhara* (pouring buttermilk over body), *pizhichil* (a specific procedure of repeatedly pouring medicine over the body)*, navarakkizhi* (a poultice made with cooked rice of a specific variety)*, navaracchoru teppu* (applying cooked rice over the skin) and *talapoticcil* (applying medicinal paste over the head) are popularly known to be part of *‘Keraliy* *Panchakarma’* techniques. Śirassekādi Vidhi also attempts to explain and analyse these procedures using the basic principles of Ayurveda. [5]

It is interesting to note that different chapters of the book begin by offering prayers to specific Gods, with interesting logics behind the choice of the Gods. The first chapter *‘Śirasseka Vidhi’*, that is *dhara* onpouring medicated fluids over the head of the patients, begins with a prayer to God Rudra who is also offered prayers by *dhāra*, which is pouring water, along with chanting of prayers. The second chapter, *‘Kayaseka Vidhi’* dealing with pouring *snehadravya* on the body of the patients, begins with a prayer to God Hari, (Vishnu), who is offered ghee and other *snehadravya* by devotees. (The author, in his commentary, explains that his prayers are to God Guruvayurappan, who is believed to be an incarnation of God Vishnu). The third chapter, *‘Pindasveda Vidhi’,* dealing with *svedana* (sudation) using boiled rice on the body, begins with a prayer to God Dhanvantari, who is offered *payasa*, a preparation of boiled rice, by devotees.

The author concludes the book by saluting his Guru, Kodungallur Cheriya Kochunni Thamburan, who is described as a famous *vaidya* as well as an artist. (he is described as *“sangutadi prasiddhan”* meaning ‘famous for his music etc.’).

The author mentions that he has intentionally avoided certain portions available in classical Ayurveda texts to avoid repetition. So, he only intends this book to be an addition to the already available fundamental texts of Ayurveda.

The first edition of the book was published by the author himself in 1929, while a second edition happened eighty years later, in 2009, by the Vaidyabhooshanam K. Raghavan Thirumulpad Foundation (VKRTF), Kerala, edited by Dr. M. Prasad.[3]

In his native village in Kerala, a Government Homoeopathy Dispensary built on the land donated by his family, is named after Vaidyan Raman Menon. But apart from that, it could be understood that Vaidyan Raman Menon, whose academic and clinical excellence is evident from the literary works available, and who was considered a prolific scholar of his time, has not gained the popularity and recognition that he deserved for the contributions made to Ayurveda.

